# Strategy towards Replacing Pork Backfat with a Linseed Oleogel in Frankfurter Sausages and Its Evaluation on Physicochemical, Nutritional, and Sensory Characteristics

**DOI:** 10.3390/foods8090366

**Published:** 2019-08-26

**Authors:** Daniel Franco, Artur J. Martins, María López-Pedrouso, Laura Purriños, Miguel A. Cerqueira, António A. Vicente, Lorenzo M. Pastrana, Carlos Zapata, José M. Lorenzo

**Affiliations:** 1 Centro Tecnológico de la Carne de Galicia, Rúa Galicia Nº 4, Parque Tecnológico de Galicia, San Cibrán das Viñas, 32900 Ourense, Spain; 2 Centre of Biological Engineering, University of Minho, Campus de Gualtar, 4710-057 Braga, Portugal; 3 Department of Zoology, Genetics and Physical Anthropology, University of Santiago de Compostela, 15872 Santiago de Compostela, Spain; 4 International Iberian Nanotechnology Laboratory, Av. Mestre José Veiga s/n, 4715-330 Braga, Portugal

**Keywords:** low fat, beeswax, cholesterol, saturated fatty acid (SFA), *n*-6/*n*-3, preference test

## Abstract

Different health institutions from western countries ha–ve recommended a diet higher in polyunsaturated fats, especially of the *n*-3 family. However, this is not a trivial task, especially for meat-processing sectors. The objective of this work was to assess the influence of replacing pork backfat with linseed oleogel on the main quality parameters of frankfurters. The frankfurters were formulated by the pork backfat replacement of 0% (control), 25% (SF-25), and 50% (SF-50), using a linseed oleogel gelled with beeswax. The determination of quality parameters (pH, colour, chemical composition, and texture parameters), the fatty acid profile, and the sensory evaluation was carried out for each batch. The fatty acid profile was substantially improved, and the saturated fatty acid (SFA) content was reduced from 35.15g/100g in control sausages to 33.95 and 32.34g/100 g in SF-25 and SF-50, respectively, and more balanced ratios *n*-6/*n*-3 were achieved. In addition, the sausages with linseed oleogel also decreased the cholesterol content from 25.08 mg/100 g in control sausages to 20.12 and 17.23 mg/100 g in SF-25 and SF-50, respectively. It may therefore be concluded that these innovative meat products are a healthier alternative. However, sensory parameters should be improved in order to increase consumer acceptability, and further research is needed.

## 1. Introduction

The consumer demand for healthy products is expected to grow in the coming years, frankfurter sausages being one of the most popular, with a large market worldwide for their convenience and price. Even though their intrinsic characteristics can vary significantly, pork frankfurters may contain up to 23% fat and 8.7% saturated fatty acids (SFA) [1], which converts them into less attractive meat products. It is a well-known fact that SFA and trans fatty acids (TFA) provide a suitable texture and juiciness in meat processed products, but they have detrimental effects on human health, such as cardiovascular disease or metabolic syndrome [2]. For this reason, the WHO recommends reducing the energy intake of total fats to less than 30% of the total daily diet and preventing SFA and TFA in the diet [3]. As animal fats are richer in SFA and TFA than those from a vegetal source, the partial replacement has become a strategic target for the meat industry in order to develop healthier meat products [4,5,6,7,8]. Indeed, vegetable oils in sausage formulations have been used for the production of low/improved fat frankfurters [9,10]. However, since vegetable oils are liquid at room temperature, this constitutes a major problem, resulting in substantial differences in texture, color, and flavor with regard to beef or pork backfat.

Trying to address these issues, food researchers have shown an increased interest in edible oleogels. The formation of oleogels requires an oleogelator that will form a network allowing the conversion of liquid oil to a solid substance, and it may also include bioactive compounds [11,12]. The properties of the oleogels are affected by the type, concentrations, and crystallization temperature of the oleogelator, by the oil medium and presence of other additives [2]. Oleogels based on canola oil and ethylcellulose as the organogelator have proven to be an alternative lipid phase in comminuted meat products [13,14], meat batters [15], and breakfast sausages [16]. However, temperatures required to dissolve ethylcellulose in oil are above 130 °C, which makes the whole process more complex. Alternatively, beeswax is certainly regarded as one of the most commercially valuable waxes, used to wrap cheese or as a food additive, which also can be used as an oleogelator. Beeswax was evaluated with different types of oils [11,17], but there is still insufficient data in meat processed products with few exceptions, such as beef burgers [18] or pâtés [19]. On the other hand, linseed oil is widely used in functional food because of its high level of α-linolenic (~55%), which is converted into long chain *n*-3 fatty acids (FA). Moreover, Fayaz et al. [20] have reported that textural properties of oleogels composed by beeswax and linseed oil were stable, showing greater firmness and stickiness than those from other oilseeds (canola, sesame, and sunflower). In addition, meat products enriched with linseed oil enhance the FA profile in health terms. Recently, Gómez–Estaca et al. [19] tested a mixture of olive, linseed, and fish oil together with beeswax to replace animal fat in pork liver pâtés, concluding that stability, texture, color, and sensory attributes were not significantly affected.

Therefore, the aim of this research was to study the effect of replacing pork backfat with different substitution levels with oleogels, elaborated using linseed oil and beeswax, on the main quality attributes of frankfurters (color, texture, and sensory properties).

## 2. Materials and Methods

### 2.1. Oleogel Elaboration

For the production of beeswax-based oleogel, a commercial linseed oil Vitaquell^®^ with 72% polyunsaturated (approx. 55% of α-linolenic), 19% monounsaturated, and 9% saturated FAs was used as the oil phase. Oleogels with 8% (*w*/*w*) gelator were produced for all the fat replacement experiments. To ensure the solubilization of beeswax in linseed oil, the gelator was dispersed under stirring at 80 °C (above wax melting point) for at least 30 min. After that period of time, the oleogels were left cooling at ambient temperature until gel formation.

### 2.2. Frankfurter Production

Three different batches (1.5 kg per batch) were produced: A control batch (CO), elaborated only with pork backfat, two batches of sausage frankfurter (SF), SF-25, and SF-50 with 25% and 50% of pork backfat replaced by oleogel, respectively. The amount of pork backfat added was 172.5 g, 129.4 g, and 86.25 g for the CO, SF-25 and SF-50, respectively, and the amount of linseed oleogel added was 43.1 g and 86.25 g for SF-25 and SF-50, respectively. These formulations were selected according to previous works developed in our lab. The other components were added in the same proportion: Pork jowl (438.8 g), pork lean (198.8 g), pork heart (240 g), water (172.5 g), ice (418.1 g), sodium caseinate (18.8 g), salt (11.2 g), and commercial mix (189.4 g). The commercial mix (Ceylamix PT-F, Laboratorios Ceylamix, Valencia, Spain) consisted of potato starch, salt, milk, and soy protein, polyphosphates (E-450i, E-452i), dextrose glutamate monosodium (E-621), sodium ascorbate (E-301), sodium, nitrite (E-250), and paprika extract (E-160c).

The meat batter was elaborated as follows. The sodium caseinate and water at 60 °C were homogenized in a Ultraturrax T25 basic (IKA-Werke, Staufen, Germany) for 2 min at a ratio of 5:1. Pork backfat or oleogel were then added and emulsified for 3 min. Once the emulsifying process was finished, the mixture was refrigerated at ambient temperature. Meat batters were elaborated by chopping the pork lean, jowl, and heart in a cutter (Cutter K30, Talsa, Talsabell S.A., Valencia, Spain) at a low speed for 1 min. Afterwards, the pork backfat or oleogel mass was added and chopped for 1 additional min. Finally, the commercial mix was included in the meat batter and mixed for 5 min. During this process, the overall temperature did not exceed 8 °C. After emulsification, each batch of meat batter was stuffed into 25 mm collagen casings and divided into sausages of 8 cm of length.

The sausages were cooked in a water bath (Marmite Mera 120 × 70, Talsa, Talsabell S.A., Valencia, Spain) at 90 °C for 20 min. The sausages were then immersed in an ice water-bath for 5 min, placed in polyethylene bags, vacuum packaged, and pasteurized at 90 °C for 30 min. Finally, the sausages were transferred to a cooler at 2 °C and stored for 24 hours until analysis. A total of 30 sausages of 100 grams (five samples per each batch × three batches × two replicates) were analyzed for different quality traits. Before analysis, sausages were minced in a blender (Moulinex, Barcelona, Spain).

### 2.3. Physicochemical Analysis

#### 2.3.1. Determination of Quality Parameters: pH, Color, and Chemical Composition

The pH value was measured using a portable pH-meter (Hanna Instruments, Eibar, Spain), equipped with a penetration probe. The color determination of sausages was assessed from fresh cut cross-sections in three different points with a portable colorimeter (Konica Minolta CM-600d, Osaka, Japan), according to CIELAB space [21]: Lightness, (L*); redness, (a*); yellowness (b*), with the next settings machine (pulsed xenon arc lamp, angle of 0° viewing angle geometry, and aperture size of 8 mm). Hue (H_ab_) and chroma (C*) were calculated from the a* and b* values according to expressions:

(1) $$C^{*}=\sqrt{{\left(a^{*}\right)}^{2}+{\left(b^{*}\right)}^{2}}\;and\;h_{ab}=acr\tan\frac{b^{*}}{a^{*}}$$

Moisture, protein, and ash were assessed following the International Organization for Standardization (ISO) recommended standards [22,23,24]. Total fat was extracted according to the American Oil Chemists’ Society (AOCS) official procedure [25], while carbohydrate contents were estimated by the difference. The quantification of total cholesterol, saponification, extraction, and identification was carried out with high performance liquid chromatography (HPLC) in the normal phase, according to Dominguez et al. [26].

#### 2.3.2. Fatty Acid Profile

The total fat was extracted from 12.5 grams of the sample and 50 milligrams were utilized to determine the FA profile. Total FAs were transesterified, following Dominguez et al. [26]. The separation and quantification of the fatty acid methyl esters (FAMEs) was performed using a gas chromatograph (GC-Agilent 6890 N; Agilent Technologies Spain, S.L., Madrid, Spain) equipped with a flame ionization detector following the chromatographic conditions described by Dominguez et al. [26]. Data of FAME profiles were expressed in grams per 100 grams of fat.

To assess the nutritional properties of sausages, the ratios polyunsaturated fatty acid (PUFA)/SFA, *n*-6/*n*-3, hypocholesterolemic/Hypercholesterolemic (h/H) ratios, the nutritional value (NV), the indexes of atherogenicity (AI), and thrombogenicity (TI) were determined. The hypocholesterolemic/Hypercholesterolemic (h/H) ratio was calculated according to Santos–Silva et al. [27]: h/H = ((sum of C18:1*n*9c, C18:2*n*6, C18:3*n*6, C18:3*n*3, C20:3*n*6, C20:4*n*6, C20:5*n*3, and C22:5*n*3)/(sum of C14:0 and C16:0)). The atherogenic index (AI) and thrombogenic index (TI) were calculated according to Ulbricht and Sauthgate [28]:

AI = [(4 × C14:0) + C16:0]/[(ΣPUFA) + (ΣMUFA)];(2)

TI = [C14:0 + C16:0 + C18:0]/[(0.5 × ΣMUFA) + (0.5 × *n*6) + (3 × *n*3) + (*n*3/*n*6)].

#### 2.3.3. Texture Profile Analysis

Sausage pieces of 1 × 1 × 2.5 cm (height × width × length) were compressed at a crosshead speed of 3.33 mm/s in a texture analyzer (TA.XTplus, Stable Micro Systems, Vienna Court, UK). The following parameters were recorded: Hardness, cohesiveness, springiness, gumminess, chewiness, and adhesiveness by compressing to 80%, using a compression probe with 19.85 cm^2^ of surface contact.

#### 2.3.4. Sensory Evaluation

A pilot consumer test was performed by 32 panelists, recruited among personnel of the Meat Technology Center. The testing was carried out in a room equipped with individual tasting booths under white light [29]. Water and bread without salt were available for rinsing the mouth between samples. Samples were cooked in a hot steam oven (CombiMaster^®^Plus, Rational, Landsberg am Lech, Germany) until a core temperature of 70 ± 2 °C was reached. The temperature was controlled using an oven thermometer inserted lengthwise into one of the sausages. After cooking, the sausages were sliced into 1.5 cm thick pieces and served hot. The serving temperature of the samples was 50 ± 2 °C. The three samples were presented to the panelists following a balanced order for each of them [30]. Panelists were asked to rank the samples according to their own preference related to appearance, odor, hardness, juiciness, taste, and global perception. They also evaluated the overall acceptability of each sample using a 7-point hedonic scale, varying from (1) “liked very much” to (7) “disliked very much”, according to Meilgaard et al. [31].

### 2.4. Statistical Analysis

All statistical analysis was performed using SPSS v. 23 package (IBM SPSS, Chicago, Illinois, USA). The effect of inclusion of oleogel at two levels (25 and 50%) was evaluated employing a mixed-model analysis of variance (ANOVA), where these traits were set as dependent variables, oleogel concentration (0, 25, and 50%) as a fixed effect, and a replicate as a random effect. The pairwise differences between least-square means were evaluated by Duncan’s test. Correlations among traits were determined by Pearson’s linear correlation coefficient.

The XLSTAT-Sensory version 2018 software was used to analyze all the sensory data. To analyze differences in the sensorial parameters evaluated by the panelist preference ranking test, the Friedman rank sum test was performed [32], using a significance level of 95% to determine whether the panelists were able to discriminate among samples. The least significant difference was used to determine whether significant differences (*p* < 0.05) existed among sausages.

Overall acceptability data from the evaluation was analyzed by means of a linear mixed model. A sample was specified as a fixed effect while a panelist was specified as a random effect. A significance level of 95% was considered in the analysis and Tukey´s test was used to separate means.

## 3. Results

### 3.1. Chemical Composition of Frankfurters

The statistical analysis showed significant differences for all evaluated parameters; however, the numerical values among batches were similar, with the exception of cholesterol content (Table 1). These outcomes can be explained by the fact that the amount of ingredients in all formulations was in fact the same, except the subcutaneous fat from pork which was replaced by the linseed oleogel. These slight observed differences could be attributed to the lack of homogeneity (backfat, jowl, lean, and heart) in the frankfurter elaboration procedure.

Differences in moisture content ranged between 55.55–56.74% for all the batches, while the protein percentage varied from 10.62 to 11.19%, resulting in slightly higher values for the control samples. In addition, the replacement with linseed oleogel had a significant (*p* < 0.05) effect on protein content. Ash content was significantly affected by replacement of pork backfat, ranging between 4.04 and 4.24%. Regarding the fat content, the differences among batches were small (18.35–20.03%), although they reached statistical (*p* < 0.05) relevance and the cholesterol amount varied significantly (*p* < 0.001) among batches, with the highest content (25.08 mg/100g) in the control sausages.

### 3.2. Fatty Acid Profile of Frankfurters

The influence of the partial replacement of pork backfat at two levels by linseed oleogel on the FA profile is shown in Table 2. As expected, this partial substitution caused a significant effect in all FAs with the exception of α-linoleic acid (C18:2*n*6c). This is due to the healthy FA profile of the linseed oleogel. However, the most predominant FAs in all batches were monosaturated fatty acid (MUFA), followed by SFA and polyunsaturated fatty acid (PUFA).

Regarding individual FA, oleic acid (C18:1*n*9c) was the most considerable FA followed by palmitic acid (C16:0), α-linoleic acid, and stearic acid (C18:0). The replacement of pork backfat by linseed oleogel reduced the SFA content from 35.15 g/100 g obtained in control sausages to 33.95 and 32.34 g/100 g in SF-25 and SF-50, respectively. The most predominant SFA was palmitic acid (C16:0), followed by stearic acid (C18:0). The palmitic content decreased significantly (*p* < 0.05) from 22.23 to 20.07 g/100 g for the control and SF-50 treatment, respectively. Moreover, stearic content showed a significant reduction from 10.92 to 10.37 g/100 g. The MUFA content was also affected by linseed replacement, presenting the highest values in the control sausages (48.06 mg/100 g). Differences are mainly due to oleic content variation along the three batches. In this sense, linseed seed is poor in this FA, causing a significant decrease in the final formulation.

Concerning PUFA, their content was significantly (*p* < 0.05) affected by linseed inclusion. Indeed, the PUFA content increased from 16.77 mg/100g for the control to 20.10 and 25.46 mg/100 g for SF-25 and SF-50, respectively. The linoleic acid did not show significant (*p* > 0.05) differences among batches; however, variations in PUFA content are directly associated to the linolenic content. This was a desired consequence, which had immediate consequences on *n*-3 PUFA content as well as in PUFA/SFA and *n*-6/*n*-3 ratios. Indeed, control sausages had the lowest PUFA/SFA ratio (0.47) and the higher *n*-6/*n*-3 (14.92). Thus, these ratios could be significantly (*p* < 0.05) increased to 0.78 and decreased to 1.61, respectively, with replacement of 50% of pork backfat by linseed oleogel.

Finally, replacing pork backfat with a linseed oleogel also had a significant effect (*p* < 0.001) on the IA and IT and on h/H. Sausages with a replacement of 25% and 50% obtained the lowest values for IA and IT (0.42 vs. 0.40 vs. 0.36; *p* < 0.001 for control, SF-25 and SF-50, respectively, in the case of IA and 0.98 vs. 0.75 vs. 0.53; *p* < 0.001 for control, SF-25 and SF-50, respectively), showing the better nutritional fatty acid profile. Regarding the h/H ratio, sausages replaced with 50% of lineseed oleogel obtained the highest values, with the highest percentage of hypocholesterolemic FA, α-linolenic, and the lowest amounts of hypercholesterolemic FA (C14:0 and C16:0). On the contrary, control sausages displayed an opposite trend in respect to the amounts of hypocholesterolemic and hypercholesterolemic FA, resulting in the lowest h/H values. However, for all sausage batches studied this ratio was higher than 2.5, which is considered as favorable.

### 3.3. Quality Parameters: pH and Color Assessment of Frankfurters

**Figure 1:**
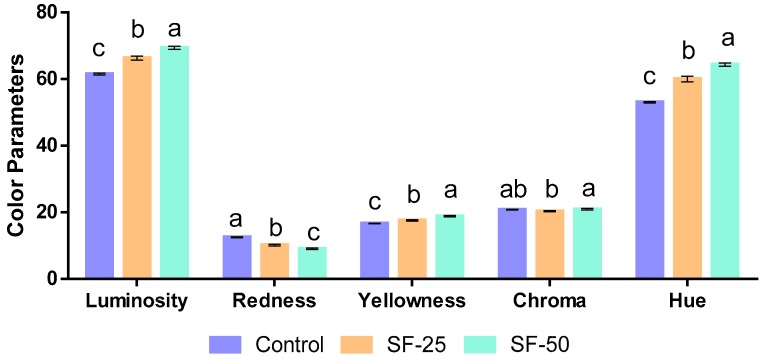
Color parameters of frankfurters, ^a–c^ Different letters indicate statistically significant differences (*p* < 0.05), (mean values ± standard deviation of 10 samples).

The pH values were significantly (*p* < 0.001) influenced by replacement with linseed oleogel, although numerical values were very similar. Regarding color parameters (L*, a*, and b*), they were significantly (*p* < 0.001) influenced by substitution of pork backfat with linseed oleogel. Specifically, luminosity and yellowness increase with the amount of linseed oleogel (L* from 61.47 to 69.37 and b* from 16.61 to 18.85 for the control and SF-50, respectively). On the contrary, redness value decreased from 12.50 to 9.06 for control and SF-50, respectively (Figure 1).

### 3.4. Texture Profile Analysis of Frankfurters

**Figure 2:**
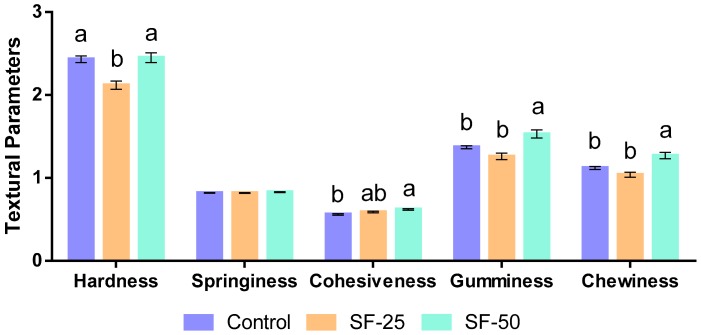
Textural parameters of frankfurters. Hardness (Kg), Springiness (mm), Gumminess (Kg), and Chewiness (kg mm), ^a–c^ Different letters indicate statistically significant differences (*p* < 0.05), (mean values ± standard deviation of 10 samples).

The textural profile analysis shows that the replacement of pork backfat with linseed oleogel led to significant variations in the following textural parameters: Hardness, cohesiveness, gumminess, and chewiness (Figure 2). Cohesiveness, gumminess, and chewiness significantly increased in SF-50, with respect to control sausages, but the differences were relatively slight.

### 3.5. Sensory Attributes of Frankfurters

Sensory analysis indicated that there were significant differences in the preference (*p* < 0.05), influenced by the replacement of pork backfat with linseed oleogel (Table 3). The appearance is significant to consumer preference and acceptability of products. Panelists showed a clear preference (*p* < 0.05) for control samples over SF-25 and SF-50 samples. Regarding color parameters, the control sample obtained the lowest yellowness value, suggesting a higher acceptability. In meat products, the yellow color is associated with rancid foods caused by lipid oxidation. A significant difference (*p* < 0.05) was detected between the control and the remaining samples for the odor and taste. The control sample was the most preferred in both cases. Hardness only showed a slightly higher value in the 25% substitution sausage without significant influence in the evaluation for ranking preference. In the case of juiciness, a significantly lower score (*p* < 0.05) showed for the SF-50, whereas no differences were detected between the control and SF-25. Finally, control samples obtained higher scores for global perception compared to other samples (86 vs. 58 and 48 for control, SF-25, and SF-50 batches, respectively).

## 4. Discussion

Among batches, variations in moisture content were slightly lower than some described by other authors on frankfurters [10,33,34]. Differences in protein content between control sausages and the other batches (SF-25 and SF-50) could be explained by the presence of small lean portions in pork backfat. Other authors have reported frankfurter sausages with protein content between 12.5–15% [10,33,34]. The range of ashes content was higher than those shown by other authors [10,33,34], who indicated values in the range 2.58 to 3.39%. These differences could be explained by variations in the amount of sodium caseinate, salt, and commercial mix among recipes of the studies.

Previous studies have noted the importance of animal fat in this type of meat products, because it plays a significant role in flavor intensity, juiciness, and tenderness. In comminuted meat products and frankfurters, values of around 26% have been found [13,35], but other lower values have been reported as 20.8% in breakfast sausage [16], 14.41% in sausages elaborated with microencapsulated fish oil [34], or even 10.3% for low-fat sausages elaborated with Konjac gel [10]. Regarding cholesterol content, it is remarkable that frankfurters formulated with linseed had the lowest cholesterol content with respect to the control sausages (25.08 vs. 17.23; *p* < 0.05), despite the fact that this result was expectable, because cholesterol is inherent to animal tissues, hence it should not be found in linseed. This is an important improvement from a nutritional point of view.

Overall, the individual FA in the following order (oleic > palmitic > linoleic > stearic) match those reported in earlier studies of frankfurters [33,34,36]. It should be noted that a noticeable SFA reduction (in the range 3.4–7.99%) was achieved as a result of the replacement of pork backfat by linseed oleogel. A similar reduction range (3–8%) was obtained with sausages containing fish oil [37]. Other authors have reported higher reductions in SFA content when replacing pork backfat with fish and/or vegetable oils, e.g., 4.5–11.8% [34] and 3–25% [38] in frankfurter sausages.

Reductions in the main SFA (palmitic and stearic acids) have important and beneficial effects on human health in accordance with the recommendations of WHO [3]. Recommendations include reducing SFA intake, due to the raise of low-density lipoprotein (LDL)-cholesterol producing atherogenic and hypercholesterolemic effects [39]. This result may be explained by the fact that linseed has a lower SFA percentage than pork backfat. These findings match those mentioned in previous reports, in regard to the fat substitution in frankfurters with vegetable oils [10,33,34].

On the other hand, lipid oxidation affects color, texture, nutritional value, taste, and aroma leading to rancidity, which is responsible for off-flavors and unacceptable taste, which are important reasons for consumer rejection [40,41]. Lipid oxidation is a rather complex process, in which unsaturated fatty acids react with molecular oxygen via free radical chain-forming peroxides [42]. The first auto-oxidation is followed by a series of secondary reactions, which lead to lipid degradation and the development of oxidative rancidity products. However, lipid oxidation (Thiobarbituric acid reactive substances values) were not determined in our sausage samples.

Variations in PUFA content, as well as PUFA/SFA and *n*-6/*n*-3 ratios, have important implications for the development of healthy meat products, as it is well known that balanced ratios of *n*-6/*n*-3 and PUFA/SFA have positive effects on human health. Effectively, excessive *n*-6 PUFA content and therefore greater *n*-6/*n*-3 ratios can result in diseases, such as cardiovascular pathology and prostate cancer, whereas an excessive increase of *n*-3 PUFA exerts suppressive effects [43]. However, western diets are increasing the amount of *n*-6 PUFA. Higher proportions of *n*-3 PUFA have been recommended by European Food Safety Authority (EFSA) [39] and Food and Agriculture Organization (FAO) [44] in order to decrease the *n*-6/*n*-3 ratio for the prevention of above-mentioned diseases. The present results agree with the findings of other studies in which the inclusion of olive and fish oils, mixture or alone, diminished the *n*-6/*n*-3 ratio in frankfurters [34] and beef burgers [45].

The color parameters of batches formulated with linseed oleogel are strongly related to color characteristics of raw ingredients used in the formulation. The increase in luminosity and yellowness may be explained by the yellow color of linseed oleogel and is associated with the amount used in the elaboration. Other authors have reported similar results in different meat products, replacing pork backfat with vegetable oils (olive, canola), such as frankfurters [34], breakfast sausages [16], pate [46], or pork patties [47]. On the contrary, Barbut et al. [16] indicated a significant decrease in the luminosity of breakfast sausages elaborated with canola oleogel. The significant (*p* < 0.001) reduction of redness values in SF-50 (9.06) and SF-25 (10.15), with respect to the control samples (12.50), seems to be consistent with the study of Lopez-Lopez et al. [9], which found that the substitution of pork backfat by olive oil decreased the redness value in frankfurters.

One of the main issues in the reformulation of meat products, such as frankfurters or sausages, is regarding textural properties, because some of these properties were quite different in new products than original products. For instance, Barbut et al. [16] indicated that the replacement of beef fat by canola oil in comminuted products led to a firmer and higher rubbery product, which is unsatisfactory. However, in our study, changes in the sausage textural profile were not remarkable, in agreement with [33], who observed no influence on the hardness or chewiness of frankfurters with olive oil replacing pork backfat. However, this issue is controversial, as the increase of hardness and firmness in frankfurters with olive oil instead of pork backfat [48,49], as well as no influence [50], has also been reported. In addition, in the present study, a negative correlation between hardness and fat content was found (*r* = −0.560; *p* < 0.01). A similar behavior was noticed for gumminess and chewiness (*r* = −0.444 and −0.428, *p* < 0.05, respectively) while adhesiveness was positive, correlated with total fat content (*r* = 0.414, *p* < 0.05).

Modifications in the formulation resulted in global perception being significantly different among samples. Thus, samples with linseed oleogel were assessed with a lower preference. The overall acceptability for sausages depends on many attributes and their interactions. Panelists showed significant differences regarding the sausages with substitution of pork backfat, with overall acceptability scores of 2.06 ± 0.17, 2.69 ± 0.19 and 3.19 ± 0.17 for CO, SF-25, and SF-50. Although the sausage control showed more acceptability than the modified sausages, the three formulations scored in the positive part of the hedonic scale used in this study, obtaining a positive acceptance. Barbut et al. [13] noticed that there may be a hardness and juiciness tipping-point based on the ethylcellulose (EC) concentration in the organogel, where frankfurters containing organogels of lower EC content resembled those prepared with beef fat (control group). In addition, they reported that organogels with higher EC concentration-yielded frankfurters that resembled the control group, potentially allowing for the custom formulation of hardness and other sensory characteristics. Organogel samples formulated with sorbitan monostearate can further help tailor textural and sensory characteristics of emulsion type meat products by adding plasticity to the organogel structure [13].

## 5. Conclusions

Linseed oleogel was successfully inserted into frankfurters in order to replace the pork backfat. The FA profile improved with the use of oleogels, the values of SFA content and the *n*-6/*n*-3 ratio reduced significantly, and the cholesterol amount also decreased significantly in the sausages with linseed oleogel. These healthier newly emerging meat products are being screened in the meat industry. However, other sensory parameters could not be substantially improved, such as color, which increased the yellowness with linseed oleogel. Moreover, cohesiveness, gumminess, and chewiness presented higher values in this new product. More research is needed to enhance the sensory properties and lipid oxidation in precooked products with linseed oleogels.

## Figures and Tables

**Table 1:** Chemical composition of a frankfurter, expressed in percentage (mean values ± standard deviation of 10 samples).

Parameter	Control	SF-25	SF-50	SEM	*p*-Value
Moisture (%)	57.55 ± 1.02 ^a^	55.98 ± 0.49 ^c^	56.74 ± 0.67^b^	0.17	*p* < 0.001
Protein (%)	11.19 ± 0.33 ^a^	10.63 ± 0.39 ^b^	10.62 ± 0.22^b^	0.07	*p* = 0.001
Ash (%)	4.24 ± 0.17 ^a^	4.05 ± 0.09 ^b^	4.04 ± 0.10^b^	0.03	*p* = 0.002
Fat (%)	18.35 ± 0.60 ^b^	20.03 ± 1.04 ^a^	18.95 ± 0.72^b^	0.19	*p* < 0.001
Carbohydrate (%)	8.65 ± 0.57 ^b^	9.30 ± 0.66 ^a^	9.63 ± 0.60^a^	0.13	*p* = 0.005
Cholesterol (mg/100 g)	25.08 ± 0.88 ^a^	20.12 ± 0.77 ^b^	17.23 ± 0.38^c^	0.87	*p* < 0.001

Saturated fatty (SF), Standard error of mean (SEM), ^a–c^ Means in the same row with different letters differing significantly (*p* < 0.05).

**Table 2:** Fatty acid profile (g/100 g of fat) of frankfurter (mean values ± standard deviation of 10 samples).

Fatty Acid	Control	SF-25	SF-50	SEM	*p*-Value
C14:0	1.26 ± 0.01 ^a^	1.27 ± 0.02 ^a^	1.16 ± 0.03 ^b^	0.01	*p* < 0.001
C16:0	22.23 ± 0.02 ^a^	21.49 ± 0.25 ^b^	20.07 ± 0.40 ^c^	0.17	*p* = 0.002
C16:1*n*7	2.27 ± 0.07 ^a^	2.28 ± 0.01 ^a^	1.95 ± 0.09 ^b^	0.03	*p* < 0.001
C17:0	0.38 ± 0.01 ^a^	0.35 ± 0.01 ^b^	0.31 ± 0.01 ^c^	0.005	*p* < 0.001
C18:0	10.92 ± 0.04 ^a^	10.47 ± 0.16 ^b^	10.37 ± 0.03 ^c^	0.04	*p* < 0.001
C18:1*n*7t	0.30 ± 0.01 ^a^	0.28 ± 0.01 ^b^	0.24 ± 0.01 ^c^	0.04	*p* < 0.001
C18:1*n*9c	40.93 ± 0.04 ^a^	39.00 ± 0.59 ^b^	36.13 ± 0.78 ^c^	0.37	*p* < 0.001
C18:1*n*7c	3.20 ± 0.01 ^a^	3.10 ± 0.03 ^b^	2.75 ± 0.10 ^c^	0.036	*p* < 0.001
C18:2*n*6c	14.31 ± 0.04	14.30 ± 0.06	14.34 ± 0.02	0.008	*p* = 0.965
C:20:0	0.15 ± 0.01 ^c^	0.16 ± 0.01 ^b^	0.17 ± 0.0 ^a^	0.002	*p* < 0.001
C18:3*n*3	0.80 ± 0.03 ^c^	4.27 ± 1.09 ^b^	9.68 ± 1.49 ^a^	0.70	*p* < 0.001
C20:2*n*6	0.68 ± 0.01 ^a^	0.62 ± 0.01 ^b^	0.54 ± 0.02 ^c^	0.01	*p* < 0.001
C20:4*n*6	0.46 ± 0.01 ^a^	0.41 ± 0.01 ^b^	0.40 ± 0.01 ^c^	0.005	*p* < 0.001
C22:5*n*3	0.09 ± 0.01 ^a^	0.08 ± 0.01 ^b^	0.07 ± 0.01 ^c^	0.001	*p* < 0.001
SFA	35.15 ± 0.07 ^a^	33.95 ± 0.41 ^b^	32.34 ± 0.45 ^c^	0.22	*p* <0.001
MUFA	48.06 ± 0.05 ^a^	45.93 ± 0.65 ^b^	42.18 ± 1.03 ^c^	0.46	*p* < 0.001
PUFA	16.77 ± 0.07 ^c^	20.10 ± 1.06 ^b^	25.46 ± 1.48 ^a^	0.69	*p* < 0.001
*n*6/*n*3	14.92 ± 0.44 ^a^	3.87 ± 2.01 ^b^	1.61 ± 0.39 ^c^	1.10	*p* < 0.001
PUFA/SFA	0.47 ± 0.01 ^c^	0.59 ± 0.04 ^b^	0.78 ± 0.05 ^a^	0.02	*p* < 0.001
IA	0.42 ± 0.01 ^a^	0.40 ± 0.01 ^b^	0.36 ± 0.01 ^c^	0.004	*p* < 0.001
IT	0.98 ± 0.01 ^a^	0.75 ± 0.07 ^b^	0.53 ± 0.05 ^c^	0.03	*p* < 0.001
h/H	2.53 ± 0.01 ^c^	2.67 ± 0.04 ^b^	2.97 ± 0.08 ^a^	0.03	*p* < 0.001

Standard error of mean (SEM), the saturated fatty acid (SFA), monounsaturated fatty acid (MUFA), polyunsaturated fatty acid (PUFA), atherogenicity (IA), thrombogenicity (IT), ^a–c^ Means in the same row with different letters differing significantly (*p* < 0.05).

**Table 3:** Preference data: Rank sums. Ranking preference: 1 the lowest, 3 the highest preferred.

Parameter	Control	SF-25	SF-50
Appearance	84 ^a^	44 ^b^	64 ^b^
Odor	86 ^a^	56 ^b^	50 ^b^
Hardness	60	74	58
Juiciness	72 ^a^	72 ^a^	48 ^b^
Taste	82 ^a^	60 ^b^	50 ^b^
Global Perception	86 ^a^	58 ^b^	48 ^b^

^a–b^ Different letters indicate statistically significant differences (*p* < 0.05).

## References

[1] USDA (2019). National Nutrient Database for Standard Reference.

[2] Jiménez-Colmenero F., Salcedo-Sandoval L., Bou R., Cofrades S., Herrero A.M., Ruiz-Capillas C. (2015). Novel applications of oil-structuring methods as a strategy to improve the fat content of meat products. Trends Food Sci. Technol..

[3] (2018). WHO. https://www.who.int/news-room/fact-sheets/detail/healthy-diet.

[4] De Carvalho F.A.L., Lorenzo J.M., Pateiro M., Bermúdez R., Purriños L., Trindade M.A. (2019). Effect of guarana (Paullinia cupana) seed and pitanga (Eugenia uniflora L.) leaf extracts on lamb burgers with fat replacement by chia oil emulsion during shelf life storage at 2 °C. Food Res. Int..

[5] Heck R.T., Saldaña E., Lorenzo J.M., Correa L.P., Fagundes M.B., Cichoski A.J., de Menezes C.R., Wagner R., Campagnol P.C.B. (2019). Hydrogelled emulsion from chia and linseed oils: A promising strategy to produce low-fat burgers with a healthier lipid profile. Meat Sci..

[6] Da Silva S.L., Amaral J., Ribeiro M., Sebastiao E., Vargas C., Frazen F., Schneider G., Lorenzo J.M., Fries L.L.M., Cichoski A.J. (2019). Fat replacement by oleogel rich in oleic acid and its impact on the technological, nutritional, oxidative, and sensory properties of Bologna-type sausages. Meat Sci..

[7] Munekata P.E.S., Dominguez R., Campagnol P.C.B., Franco D., Trindade M.A., Lorenzo J.M. (2017). Effect of natural antioxidants on physicochemical properties and lipid stability of pork liver pâté manufactured with healthy oils during refrigerated storage. J. Food Sci. Technol..

[8] Heck R.T., Guidetti R., Etchepare M.A., dos Santos L.A.A., Cichoski A.J., Ragagnin C., Smanioto J., Lorenzo J.M., Wagner R., Campagnol P.C.B. (2017). Is it possible to produce a low-fat burger with a healthy n-6/n-3 PUFA ratio without affecting the technological and sensory properties?. Meat Sci..

[9] López-López I., Cofrades S., Jiménez-Colmenero F. (2009). Low-fat frankfurters enriched with n-3 PUFA and edible seaweed: Effects of olive oil and chilled storage on physicochemical, sensory and microbial characteristics. Meat Sci..

[10] Salcedo-Sandoval L., Cofrades S., Pérez C.R.C., Solas M.T., Jiménez-Colmenero F. (2013). Healthier oils stabilized in konjac matrix as fat replacers in n-3 PUFA enriched frankfurters. Meat Sci..

[11] Patel A.R., Dewettinck K. (2016). Edible oil structuring: An overview and recent updates. Food Funct..

[12] Martins A.J., Vicente A.A., Cunha R.L., Cerqueira M.A. (2018). Edible oleogels: An opportunity for fat replacement in foods. Food Funct..

[13] Barbut S., Wood J., Marangoni A. (2016). Potential use of organogels to replace animal fat in comminuted meat products. Meat Sci..

[14] Agregán R., Barba F.J., Gavahian M., Franco D., Khaneghah A.M., Carballo J., Ferreira I.C.F.R., Barreto A.C.S., Lorenzo J.M. (2019). Fucus vesiculosus extracts as natural antioxidants for improving the physicochemical properties and shelf life of pork patties formulated with oleogels. J. Sci. Food Agric..

[15] Zetzl A.K., Marangoni A.G., Barbut S. (2012). Mechanical properties of ethylcellulose oleogels and their potential for saturated fat reduction in frankfurters. Food Funct..

[16] Barbut S., Wood J., Marangoni A. (2016). Quality effects of using organogels in breakfast sausage. Meat Sci..

[17] Martins A.J., Cerqueira M.A., Fasolin L.H., Cunha R.L., Vicente A.A. (2016). Beeswax organogels: Influence of gelator concentration and oil type in the gelation process. Food Res. Int..

[18] Moghtadaei M., Soltanizadeh N., Goli S.A.H. (2018). Production of sesame oil oleogels based on beeswax and application as partial substitutes of animal fat in beef burger. Food Res. Int..

[19] Gómez-Estaca J., Herrero A.M., Herranz B., Álvarez M.D., Jiménez-Colmenero F., Cofrades S. (2019). Characterization of ethyl cellulose and beeswax oleogels and their suitability as fat replacers in healthier lipid pâtés development. Food Hydrocoll..

[20] Fayaz G., Goli S.A.H., Kadivar M. (2017). A Novel Propolis Wax Based Organogel: Effect of Oil Type on Its Formation, Crystal Structure and Thermal Properties. J. Am. Oil Chem. Soc..

[21] CIE (1978). International Commission on Illumination, Recommendations on Uniform Color Spaces, Color Difference Equations, Psychometric Color Terms.

[22] ISO 1442 (1997). Determination of Moisture Content.

[23] ISO 937 (1978). Determination of Nitrogen Content.

[24] ISO 936 (1998). Determination of Ash Content.

[25] AOCS (2005). AOCS Official Procedure Am 5-04. Rapid Determination of Oil/Fat Utilizing High Temperature Solvent Extraction.

[26] Dominguez R., Crecente S., Borrajo P., Agregán R., Lorenzo J.M. (2015). Effect of slaughter age on foal carcass traits and meat quality. Animal.

[27] Santos-Silva J., Bessa R.J.B., Santos-Silva F. (2002). Effect of genotype, feeding system and slaughter weight on the quality of light lambs. II. Fatty acid composition of meat. Livest. Prod. Sci..

[28] Ulbricht T.L.V., Southgate D.A.T. (1991). Coronary heart disease: Seven dietary factors. Lancet.

[29] ISO 8589 (2007). Sensory Analysis-General Guidance for the Design of Test Rooms.

[30] Macfie H.J., Bratchell N., Greenhoff K., Vallis L. (1989). Designs to balance the effect of order presentation and first-order carry-over effects in hall tests. J. Sens. Stud..

[31] Meilgaard M., Civille G.V., Carr B.T. (2006). Sensory Evaluation Techniques.

[32] ISO 8587 (2006). Sensory Analysis-Methodology-Ranking.

[33] Delgado-Pando G., Cofrades S., Ruiz-Capillas C., Jiménez-Colmenero F. (2010). Healthier lipid combination as functional ingredient influencing sensory and technological properties of low-fat frankfurters. Eur. J. Lipid Sci. Technol..

[34] Dominguez R., Pateiro M., Agregán R., Lorenzo J.M. (2017). Effect of the partial replacement of pork backfat by microencapsulated fish oil or mixed fish and olive oil on the quality of frankfurter type sausage. J. Food Sci. Technol..

[35] Álvarez D., Delles R.M., Xiong Y.L., Castillo M., Payne F.A., Laencina J. (2011). Influence of canola-olive oils, rice bran and walnut on functionality and emulsion stability of frankfurters. LWT-Food Sci. Technol..

[36] Ayo J., Carballo J., Serrano J., Olmedilla-Alonso B., Ruiz-Capillas C., Jiménez-Colmenero F. (2007). Effect of total replacement of pork backfat with walnut on the nutritional profile of frankfurters. Meat Sci..

[37] Josquin N.M., Linssen J.P., Houben J.H. (2012). Quality characteristics of Dutch-style fermented sausages manufactured with partial replacement of pork back-fat with pure, pre-emulsified or encapsulated fish oil. Meat Sci..

[38] Choi Y.S., Choi J.H., Han D.J., Kim H.Y., Lee M.A., Jeong J.Y., Kim C.J. (2010). Effects of replacing pork back fat with vegetable oils and rice bran fiber on the quality of reduced-fat frankfurters. Meat Sci..

[39] EFSA (2010). Scientific opinion on dietary reference values for fats, including saturated fatty acids, polyunsaturated fatty acids, monounsaturated fatty acids, trans fatty acids, and cholesterol. EFSA J..

[40] Domínguez R., Gómez M., Fonseca S., Lorenzo J.M. (2014). Influence of thermal treatment on formation of volatile compounds, cooking loss and lipid oxidation in foal meat. LWT-Food Sci. Technol..

[41] Cossignani L., Giua L., Simonetti M.S., Blasi F. (2014). Volatile compounds as indicators of conjugated and unconjugated linoleic acid thermal oxidation. Eur. J. Lipid Sci. Technol..

[42] Giua L., Blasi F., Simonetti M.S., Cossignani L. (2013). Oxidative modifications of conjugated and unconjugated linoleic acid during heating. Food Chem..

[43] Williams C.D., Whitley B.M., Hoyo C., Grant D.J., Iraggi J.D., Newman K.A., Freedland S.J. (2011). A high ratio of dietary n-6/n-3 polyunsaturated fatty acids is associated with increased risk of prostate cancer. Nutr. Res..

[44] FAO (2010). Fats and Fatty Acids in Human Nutrition: Report of an Expert Consultation.

[45] Keenan D.F., Resconi V.C., Smyth T.J., Botinestean C., Lefranc C., Kerry J.P., Hamill R.M. (2015). The effect of partial-fat substitutions with encapsulated and unencapsulated fish oils on the technological and eating quality of beef burgers over storage. Meat Sci..

[46] Morales-Irigoyen E.E., Severiano-Pérez P., Rodriguez-Huezo M.E., Totosaus A. (2012). Textural, physicochemical and sensory properties compensation of fat replacing in pork liver pate incorporating emulsified canola oil. Food Sci. Technol. Int..

[47] Rodríguez-Carpena J.G., Morcuende D., Estévez M. (2012). Avocado, sunflower and olive oils as replacers of pork back-fat in burger patties: Effect on lipid composition, oxidative stability and quality traits. Meat Sci..

[48] Paneras E.D., Bloukas J.G. (1994). Vegetable-oils replace pork backfat for low-fat frankfurters. J. Food Sci..

[49] Paneras E.D., Bloukas J.G., Filis D.G. (1998). Production of low-fat frankfurters with vegetable oils following the dietary guidelines for fatty acids. J. Muscle Foods.

[50] Bloukas J.G., Paneras E.D. (1993). Substituting olive oil for pork backfat affects quality of low-fat frankfurters. J. Food Sci..

